# Effects of Different Pulmonary Vasodilators on Arterial Saturation in a Model of Pulmonary Hypertension

**DOI:** 10.1371/journal.pone.0073502

**Published:** 2013-08-28

**Authors:** Eva Maria Becker, Johannes-Peter Stasch, Martin Bechem, Jörg Keldenich, Alexandra Klipp, Katja Schaefer, Hannes-Friedrich Ulbrich, Hubert Truebel

**Affiliations:** 1 Bayer Pharma AG, Cardiology Research, Wuppertal, Germany; 2 University Witten/Herdecke, Fakultät für Gesundheit, Witten, Germany; 3 Bayer Pharma AG, Research and Industrial Statistics, Berlin, Germany; 4 Institute of Pharmacy, Martin Luther University Halle, Germany; University of Southampton, United Kingdom

## Abstract

**Background:**

Approved therapies for pulmonary arterial hypertension can induce oxygen desaturation when administered to patients with secondary forms of pulmonary hypertension (PH), probably due to an increase in ventilation/perfusion mismatch. Thus, so far these treatments have largely failed in secondary forms of PH.

**Methods:**

We established an animal model of heterogeneous lung ventilation to evaluate the desaturation potential of mechanistically distinct vasoactive drugs launched or currently in clinical development for the treatment of PH. Single-lung ventilation was induced in five groups (N = 6) of anesthetized minipigs (7 weeks, 4 to 5 kg BW), and their hemodynamic parameters were monitored before and after intravenous injection of control (vehicle only), endothelin antagonist (bosentan; 0.3, 1, 3, 10 mg/kg), phosphodiesterase type 5 inhibitor (sildenafil; 3, 10, 30, 100 µg/kg), and soluble guanylate cyclase stimulators (BAY 41–8543 and riociguat; 1, 3, 10, 30 µg/kg). Cumulative doses were administered before successive unilateral ventilation cycles. The doses were chosen to achieve equal effect on blood pressure by the different pharmacologic principles.

**Results:**

Single-lung ventilation resulted in transient increases in mean pulmonary artery pressure (mPAP) and desaturation. In contrast to control, all drugs dose-dependently decreased hypoxic mPAP (a positive treatment effect) and increased area under the arterial hemoglobin saturation curve (unwanted desaturation effect). Riociguat and bosentan reduced hypoxic mPAP to the greatest extent, while the soluble guanylate cyclase stimulators riociguat and BAY 41–8543 lowered arterial oxygen saturation of hemoglobin the least.

**Conclusions:**

Future investigations will be required to confirm these findings in clinical settings.

## Introduction

In order to supply the body with sufficient oxygen, the degree of pulmonary perfusion (Q′) is matched to the respective level of ventilation (V′) in the sublobar regions of the lung [1]. This homeostatic process of respiratory physiology has been known since the 1960s when physiologists studied the relation of oxygen (O_2_) uptake and carbon dioxide (CO_2_) removal under different conditions [2]. Under physiologic conditions, blood is distributed to areas of the lung that receive adequate ventilation and is “shunted” away from diseased lung tissue where ventilation is impaired [3]. This conserved phenomenon, which is based on hypoxic pulmonary vasoconstriction (HPV), occurs in pulmonary arteriolar smooth muscle cells (PASMCs) and has a central role in the matching of ventilation and pulmonary blood flow. HPV can be adversely affected by disease states and by pharmacotherapies, leading to V′/Q′ mismatch (VQM). VQM, the most common reason for gas exchange abnormalities in pulmonary diseases [1], [3], is characterized by a decrease in arterial oxygen partial pressure (paO_2_), arterial oxygen saturation of hemoglobin (SaO_2_; hypoxemia), and tissue O_2_ delivery.

Mild, moderate, and severe hypoxemias are features of some types of pulmonary hypertension (PH), which may be related to VQM [4], [5]. Recently, the treatment of chronic obstructive pulmonary disease with the endothelin receptor antagonist bosentan or the phosphodiesterase type-5 (PDE5) inhibitor sildenafil was associated with aggravation of VQM [6], [7] necessitating thorough therapeutic assessment and monitoring to avoid hypoxemia. In fact, aggravation of VQM-related desaturation likely explains why current treatment options approved for pulmonary arterial hypertension (PAH) have largely failed in secondary forms of PH [8].

Soluble guanylate cyclase (sGC) stimulators not only stimulate sGC directly but also increase the sensitivity of sGC to low levels of nitric oxide (NO), the endogenous stimulator of sGC [9]. This dual mechanism of action may be beneficial in several forms of secondary PH, in which there is severe endothelial dysfunction due to reduced endogenous NO synthesis [10]. In this regard, sGC stimulators have a different mechanism of action to PDE5 inhibitors, which depend on basal NO levels to produce an increase in cyclic guanosine monophosphate (cGMP) levels.

In order to avoid VQM with new pharmacotherapies for PH, it is important to assess their potential to cause desaturation as a “safety biomarker.” As shown in clinical settings, drugs with a vasodilatory potential have a greater propensity for VQM compared with anti-inflammatory drugs [8]. Therefore, oxygenation as a safety biomarker needs to be closely followed in all patients at risk [11]. Using a preclinical animal model mimicking the heterogeneous ventilation perfusion patterns in the lung of patients with secondary PH forms, we evaluated the “desaturation-potential” of bosentan, sildenafil, and the sGC stimulators BAY 41–8543 and riociguat (currently in clinical development for PAH and chronic thromboembolic pulmonary hypertension [CTEPH]). These drugs were chosen as they represent examples of vasodilators with the three mechanisms of action presently used or studied for oral treatment of different PH forms.

## Materials and Methods

### Ethics statement

All study procedures conformed to national legislation (dt. Tierschutzgesetz v. 18.05.2006) and EU directives (86/609) for the use of animals for scientific purposes and were approved by the institutional animal care office of Bayer AG and by the competent regional authority (LANUV NRW, permit no. 8.87–50.10.44.08.266, date of approval 14/11/2008). The study was performed according to the German animal laws under the surveillance of animal management Bayer Schering Pharma, Wuppertal, Germany. Sedation was performed by intramuscular administration of ketamine (25 mg/kg, Ketavet®, Pfizer, Germany) and azaperone (10 mg/kg; Stresnil®, Janssen, Germany). Anesthesia was introduced by intravenous administration of ketamine (1.9 mg/kg) and midazolam (0.28 mg/kg, Dormicum®, Roche, Germany) and maintained by intravenous infusion of ketamine (approximately 18 mg/kg/hour) and midazolam (approximately 2.7 mg/kg/hour) in combination with pancuroniumbromide (approximately 0.2 mg/kg/hour, Pancuronium Organon®, Essex Pharma, Germany) as detailed in the Study Design section. After the experiment, under anesthesia animals were sacrificed by intravenous injection of 4 ml T61 injection solution (5 mg tetracaine hydrochloride/50 mg mebezonium iodide/200 mg embutramide in 1 ml).

### Study design

Seven-week-old Göttingen minipigs (Ellegaard Minipigs, Dalmose, Denmark) weighing 4 to 5 kg were sedated by intramuscular administration of ketamine (25 mg/kg, Ketavet®, Pfizer, Berlin, Germany) and azaperone (10 mg/kg, Stresnil®, Janssen, Neuss, Germany). Anesthesia was introduced by intravenous administration of ketamine (1.9 mg/kg) and midazolam (0.28 mg/kg, Dormicum®, Roche, Berlin, Germany) and maintained by intravenous infusion of ketamine (approximately 18 mg/kg/h) and midazolam (approximately 2.7 mg/kg/h), given in combination with pancuroniumbromide (approximately 0.2 mg/kg/h, Pancuronium Organon®, Essex Pharma, Munich, Germany) in order to suppress spontaneous breathing and ensure stable hemodynamic and experimental conditions. Isotonic saline (10 ml/kg/h) was given for fluid maintenance. After induction of anesthesia, the animals were placed in a supine position so that a tracheotomy could be performed and a tracheal tube (I.D. 4.5 mm, Kruuse, Langeskov, Denmark) inserted. The ventilator (Sulla 808 V-D, Dräger, Lübeck, Germany) used a FiO_2_ of 40% and was adjusted to give a positive end-expiratory pressure of 5 cm H_2_O with a tidal volume of 50–60 mL at a frequency of 30 breaths per minute. A 4 French Berman Angiographic Balloon Catheter (Arrow International, Reading, PA, US) was introduced via the right internal jugular vein to measure pulmonary artery pressure (PAP). The left carotid artery was cannulated to measure arterial pressure (BP). A 3 French Micro-Tip Catheter (Föhr Medical Instruments, Seeheim-Ober Beerbach, Germany) was introduced via the left carotid artery to measure left ventricle pressure (LVP) and heart rate (HR). The left jugular vein was cannulated to measure central venous pressure (CVP). A 4 French Oximetry Catheter (Edwards Lifesciences, Irvine, CA, US) was placed into the left femoral artery and connected to the Vigilance Monitor (Edwards Lifesciences, Irvine, CA, US) for measurement of the arterial oxygen saturation of hemoglobin (SaO_2_). To measure continuous cardiac output (CCO), a 4 French thermodilution catheter (Pulsion Medical Systems, Munich, Germany) was placed into the right femoral artery and connected to the PiCCO2 (Pulsion Medical Systems, Munich, Germany). Multilumen venous catheters (BD Medical Systems, Singapore, Singapore) were placed into both femoral veins for administration of anesthesia, test compounds, fluid substitution, and regular blood sampling. Cardiovascular parameters were collected via a PoNeMah acquisition and analysis system (Data Sciences International, St. Paul, MN, US) through combitrans transducers (Combitrans, B. Braun, Melsungen, Germany) and Gould transducers (series 6600): mean values of all parameters were sampled during stable intervals of at least 1 (peak values) and 3 min (baseline). Blood gases were determined 3 min after the start of each unilateral broncho-occlusion (Stat Profile pHOx plus L; Nova Biomedical, Waltham, MA, US).

Right-sided single-lung ventilation was induced by advancing the tracheal tube into the right main bronchus followed by inflation of the cuff balloon. Tube placement into the right-sided main bronchus was confirmed by auscultation. In each animal, two cycles of 10 min of unilateral ventilation were followed by 30 min of bilateral ventilation without pharmacologic intervention to verify reproducibility of unilateral ventilation. Following these control cycles, each animal underwent five repetitive rounds of unilateral ventilation (10 min) starting with a vehicle reference cycle followed by four intervention cycles. Each of these phases was followed by 30 min of regular bilateral ventilation. Hemodynamic parameters (e.g., mPAP, BP, HR, CCO, and SaO_2_) were monitored continuously.

**Figure 1:**
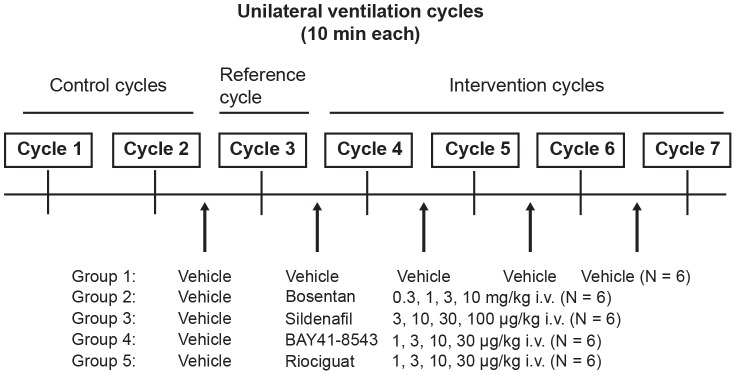
Experimental set up. Seven unilateral ventilation cycles (10 min) were performed per animal. Two cycles were performed without pharmacologic intervention to verify reproducibility of unilateral ventilation. Drugs were then applied in escalating doses (intravenous bolus injections) (N = 6 animals per group).

In five separate groups of six animals, we compared vehicle control (composition described below), bosentan (0.3, 1, 3, 10 mg/kg), sildenafil (3, 10, 30, 100 µg/kg), BAY 41–8543 (1, 3, 10, 30 µg/kg), and riociguat (1, 3, 10, 30 µg/kg). These doses were chosen to achieve similar BP reduction across each active treatment group; for each compound, an exponential dose increase spanning four orders of magnitude was administered. Cumulative doses of vasoactive compounds were applied 15 min before successive unilateral ventilation, and effects on oxygenation parameters (e.g., area under the SaO_2_ curve = AUC_SaO2_) as well as hemodynamic parameters were compared with vehicle conditions as shown in Fig. 1. Each bolus injection was performed 15 min before the unilateral ventilation cycle to ensure stable hemodynamic conditions and adequate drug distribution. All drugs were dissolved in vehicle, a mixture of dimethyl sulfoxide (Sigma-Aldrich, St. Louis, MO, US), Transcutol® (Sigma-Aldrich, St. Louis, MO, US), and PEG400 (Merck, Darmstadt, Germany) in a ratio of 1∶49.5∶49.5% and administrated as an intravenous bolus over 1–2 min at a volume of 250 µL/kg.

### Bioanalytics

In mimicking experiments for all four compounds (Supplementary Figure S1) plasma samples were taken 30 min after cumulative dosing (at least N = 3 for every dose) and processed in the same way: an aliquot of plasma was spiked with an excess amount of acetonitrile to precipitate proteins and extract the compounds from their matrix. After centrifugation, the supernatant was ready for analysis. Calibration samples with defined amounts of compound added to native plasma of the test species were handled in the same way. For each compound, a specific liquid chromatography linked to tandem mass spectrometry (LC/MS-MS) method for quantification was available. In the LC/MS-MS run for the specific compound the supernatants for plasma samples and calibration samples were measured together in a predefined sequence according to their expected concentration. With the aid of the calibration samples, a calibration curve was established and the concentrations of the plasma samples were evaluated from this curve. Thirty minutes was considered an appropriate post-administration sampling interval, as plasma-concentration time curves for these drugs decline relatively slowly. The target range for human concentrations was: bosentan 500–1000 µg/L; sildenafil 50–150 µg/L; riociguat 70–200 µg/L; BAY 41–8543, experimental compound, human concentration not known. The target range was intended to induce the same blood pressure effects with these compounds at the highest dose.

### Statistics

Animals were evaluated repeatedly at the end of each of the seven consecutive time periods (or cycles, Fig. 1). Cycles 1 and 2 were intended to prove stable baseline reactions and no vehicle solution was applied during these cycles; only animals for which this reproducibility was confirmed were used in the experiments. Cycle 3 measurements (after application of the respective vehicle to all animals) were animal-specific baseline values. Measurements from Cycles 4 through 7 were standardized by the Cycle 3 values, giving the parameter and animal specific relative changes from baseline (% change value). Statistical analyses were based on these values. Since% change values by definition have to be larger than –100%, to assume a Gaussian (normal) distribution for them is not reasonable. Therefore, a log transformation (for any% change value 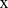 the transformed value 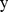 is calculated by 
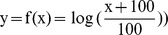
 was applied before any statistical analysis. All derived values (like means or confidence bounds) were re-transformed to the original% change scale.

Univariate% change values for all treatment groups were analyzed by analysis of variance for repeated measurements, with autoregressive of order 1 (AR[1]) covariance structure for the time (cycles) component and common variability for each group and cycle. AR(1) was appropriate since within-animal covariance was expected to decrease with the measurements being farther away.

The joint analysis of% changes in mPAP and increases in AUC_SaO2_ as the co-primary endpoints was completed by a composite covariance model [12], with AR(1) covariance structure for the time component and common variability for each group and cycle. The correlation between mPAP and increase in AUC_SaO2_ was estimated without further restrictions. All calculations were performed using SAS® version 9.2 (SAS Institute Inc., Cary, NC, USA). Graphs of the data were derived showing the rescaled mean; standard error of the mean values are based on the joint variability. No multiplicity adjustments were made.

## Results

**Figure 2:**
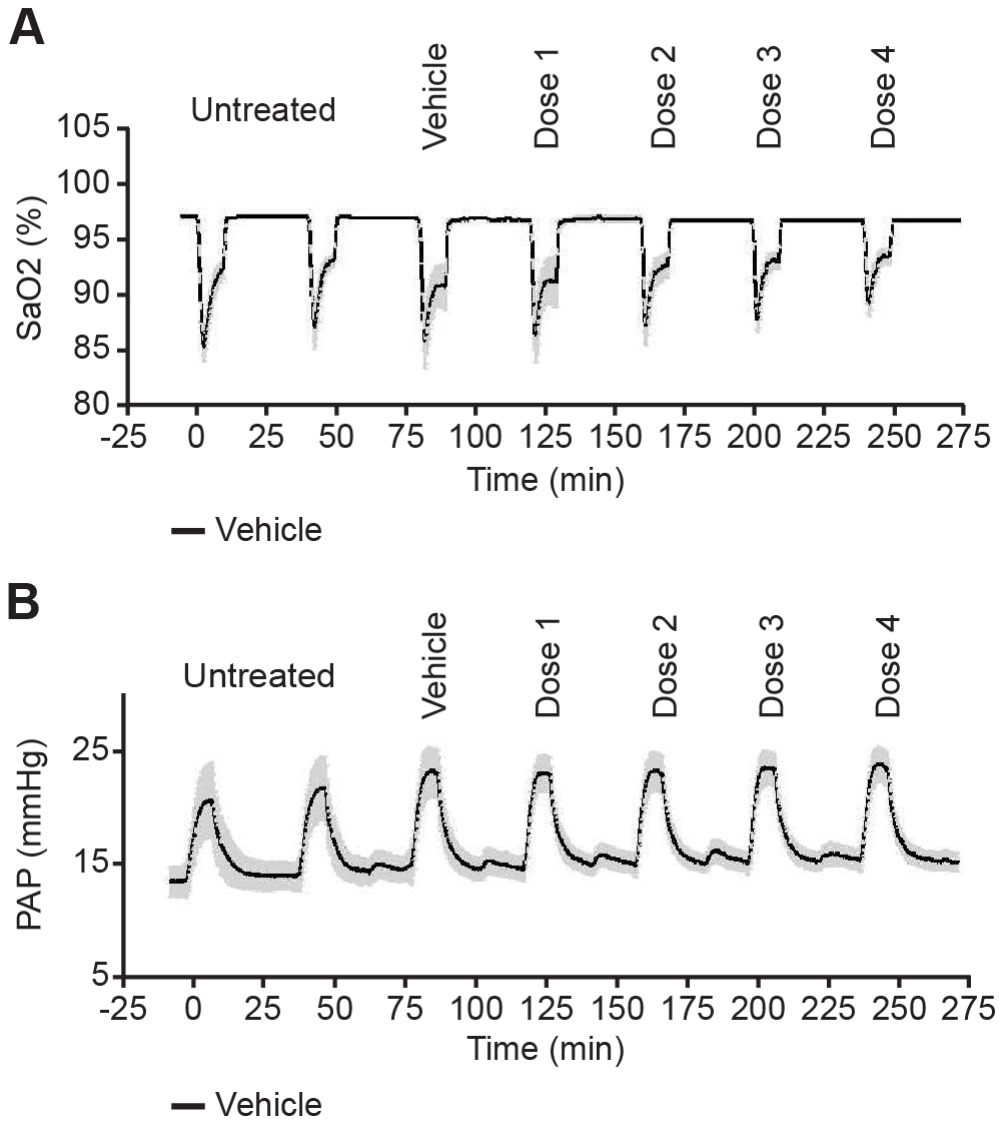
Time course of SaO_2_ saturation and mPAP. Time course of SaO_2_ saturation (A) and mPAP (B) in vehicle-treated animals (mean ± SEM, N = 6). mPAP, mean pulmonary artery pressure; SaO_2_, arterial oxygen saturation of hemoglobin; SEM, standard error of the mean.

**Figure 3:**
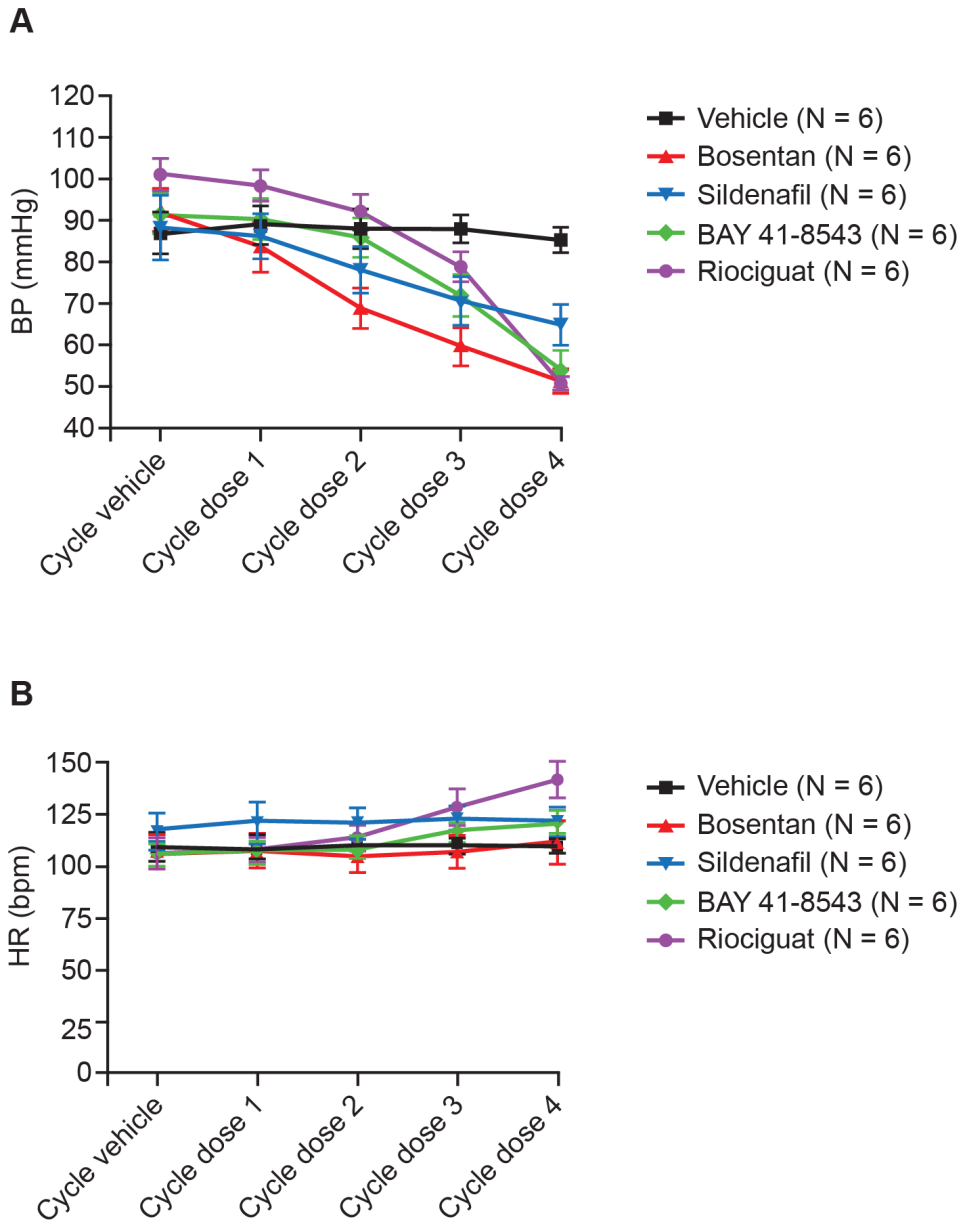
Changes in BP and HR. Changes in BP and HR during the course of the experiment of different groups are shown after each unilateral ventilation cycle (mean ± SEM, N = 6). BP, blood pressure; HR, heart rate; SEM, standard error of the mean.

In vehicle-treated control animals repetitive 10-min cycles of unilateral lung ventilation cycles resulted in reproducible increases in mPAP accompanied by decreases in SaO_2_ (Fig. 2). HR and systemic BP were stable during the entire course of the experiment (Fig. 3). During this time, vehicle-treated control animals demonstrated a trend towards a small decrease in SaO_2_ and a small increase in mPAP during the later unilateral ventilation cycles.

All of the vasodilators produced a dose-dependent decrease in BP, with bosentan being more effective at lower doses compared with riociguat and BAY 41–8543 (Fig. 3). At the highest dose tested, both sGC stimulators, BAY 41–8543 and riociguat were similar or slightly more effective at lowering BP than bosentan, whereas the PDE5 inhibitor sildenafil was less effective.

**Figure 4:**
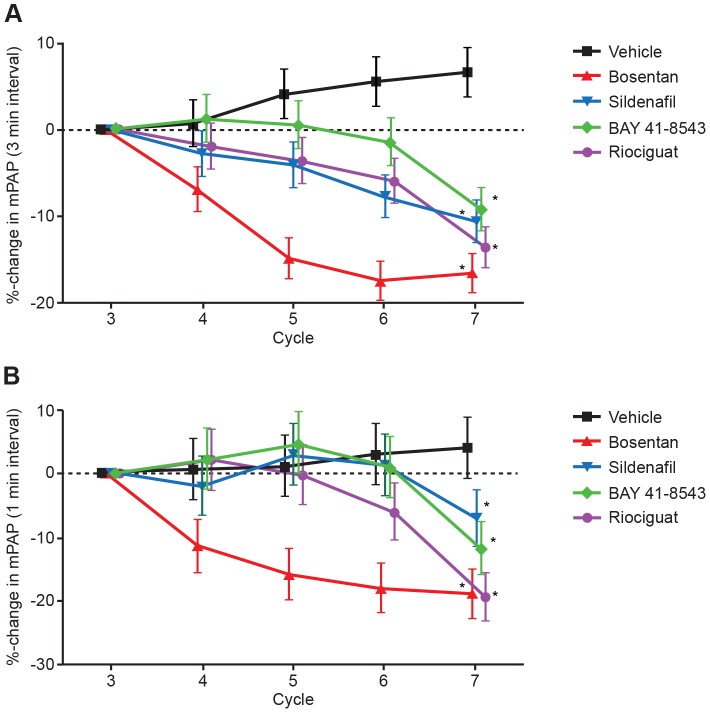
Percentage changes in mPAP. Percentage changes in mPAP under (A) normal ventilation (3-min observation interval) and (B) the peak of hypoxia induced PAP increase (1-min observation interval) during univentilation cycles versus representative vehicle cycle for each group (mean ± SEM, N = 6). mPAP, mean pulmonary artery pressure; SEM, standard error of the mean. *p<0.05 in treatment group development over time as compared with vehicle.

All the vasodilators induced a dose-dependent decrease in mPAP versus vehicle-treated animals under normal ventilation (Fig. 4A). During unilateral ventilation cycles hypoxia-induced increases in mPAP were reduced in a dose-dependent manner by the different drugs. Bosentan was most effective in lowering mPAP, then riociguat (Fig. 4B). A small increase of about 5% was observed in the vehicle treated animals. Note that the% changes represent the increase in baseline value between Cycle 3 and the following cycles, as well as the peak of Cycle 3 and the peak of the following cycles.

**Figure 5:**
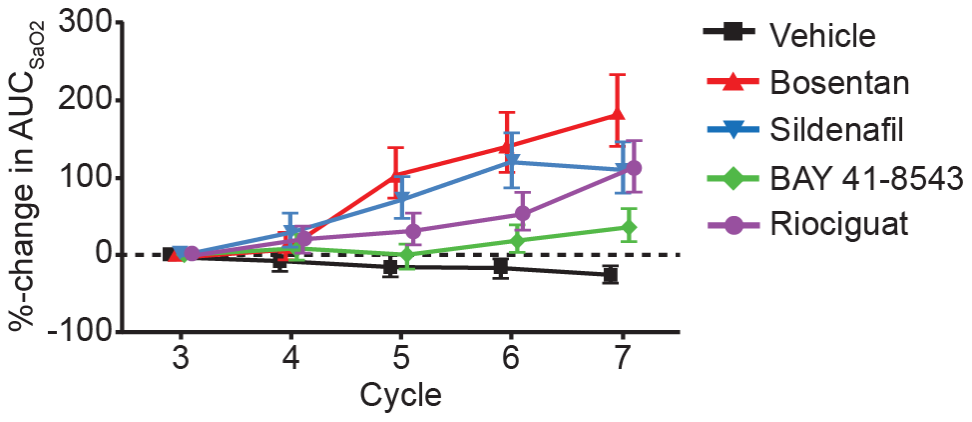
Relative change of AUC_SaO2_ versus representative vehicle cycles. Relative change of the desaturation area (AUC_SaO2_) versus representative vehicle cycles in each animal as mean ± SEM, N = 6. AUC_SaO2_, area under the SaO_2_ curve; SaO_2_, arterial oxygen saturation of hemoglobin; SEM, standard error of the mean. No significant differences were seen between treatment group development over time as compared with vehicle.

All of the vasodilators dose-dependently enhanced desaturation during unilateral ventilation cycles relative to control, as shown by the increase in the desaturation area (Fig. 5) with the sGC stimulators eliciting a lesser desaturation effect.

**Figure 6:**
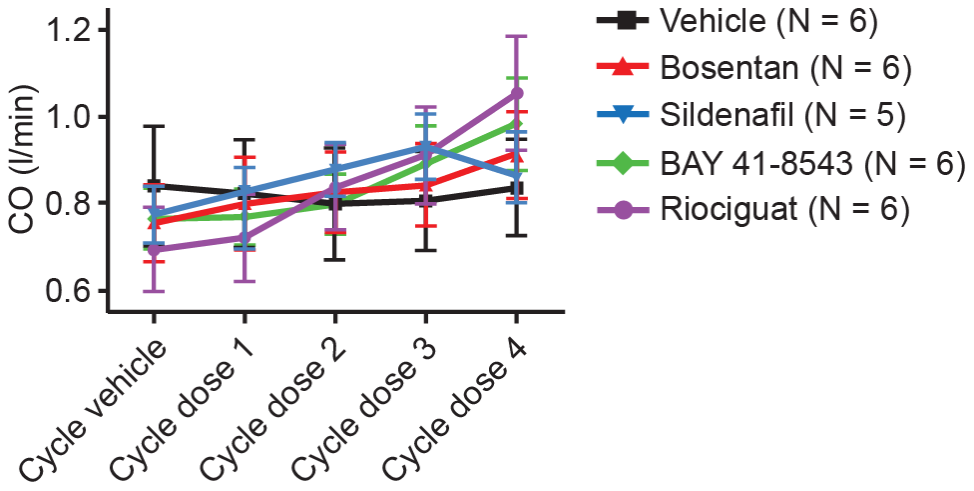
CO following each univentilation cycle. CO following each univentilation cycle, shown as mean values ± SEM out of six animals. CO, cardiac output; SEM, standard error of the mean.

**Figure 7:**
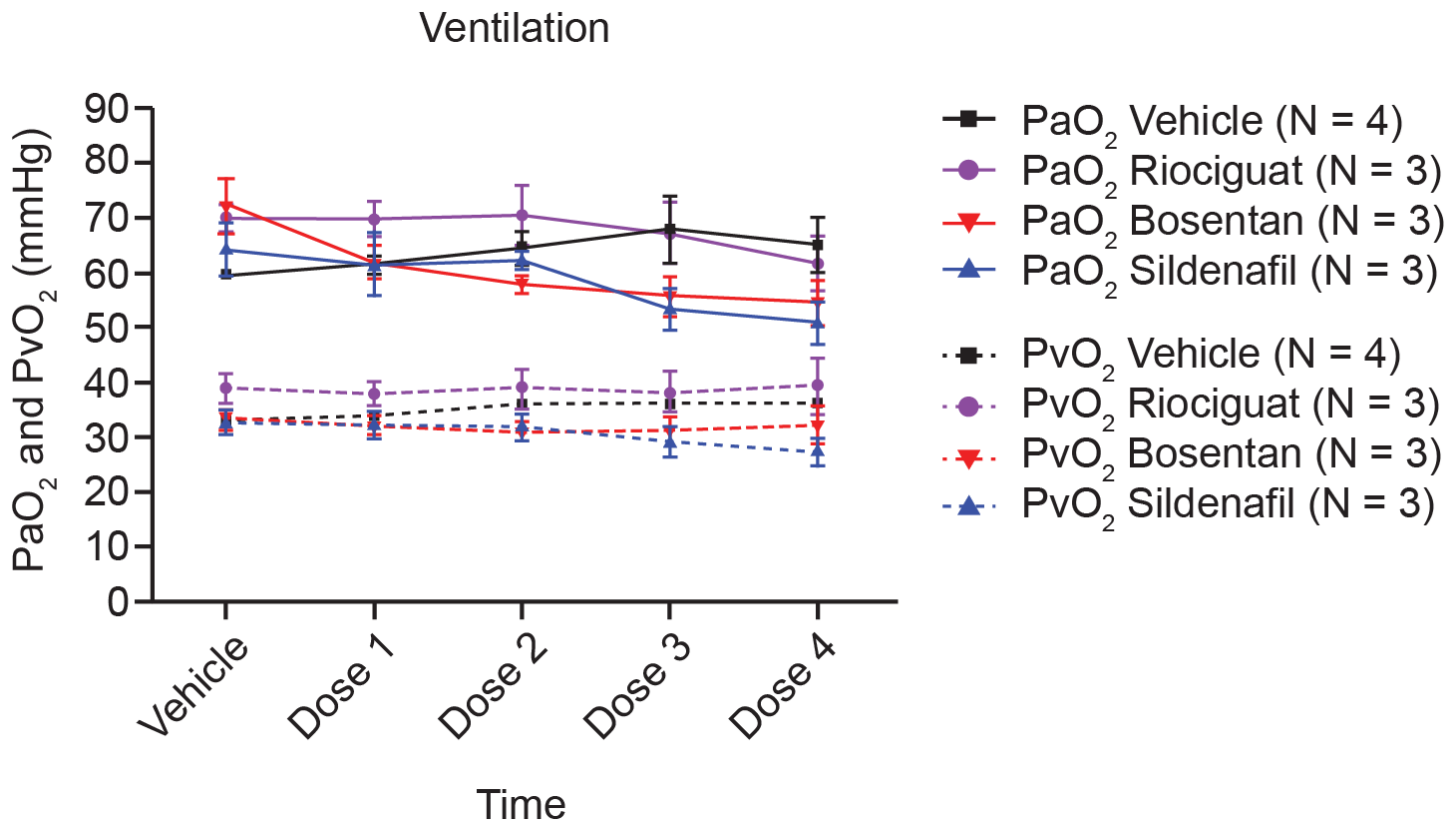
PaO_2_ and PvO_2_ after each univentilation cycle. Arterial (PaO_2_) as well as venous (PvO_2_) partial oxygen pressure of four different groups are shown after each univentilation cycle. Shown are mean values ± SEM out of three to four animals. SEM, standard error of the mean.

Compared with control, all the vasodilators showed a similar increase in CO despite their distinct mechanisms of action (Fig. 6). Nevertheless, there was no significant difference detected regarding cardiac output (CO) between drug-treated animals and control animals. In a satellite study, arterial and venous O_2_ partial pressures were measured during the experiment to avoid differences between groups driven by differences in partial O_2_ pressure leading to the observed differences in the desaturation areas. We did not observe statistically significant differences in arterial or venous partial O_2_ pressure between the different groups at baseline or during the course of the experiment (Fig. 7).

**Figure 8:**
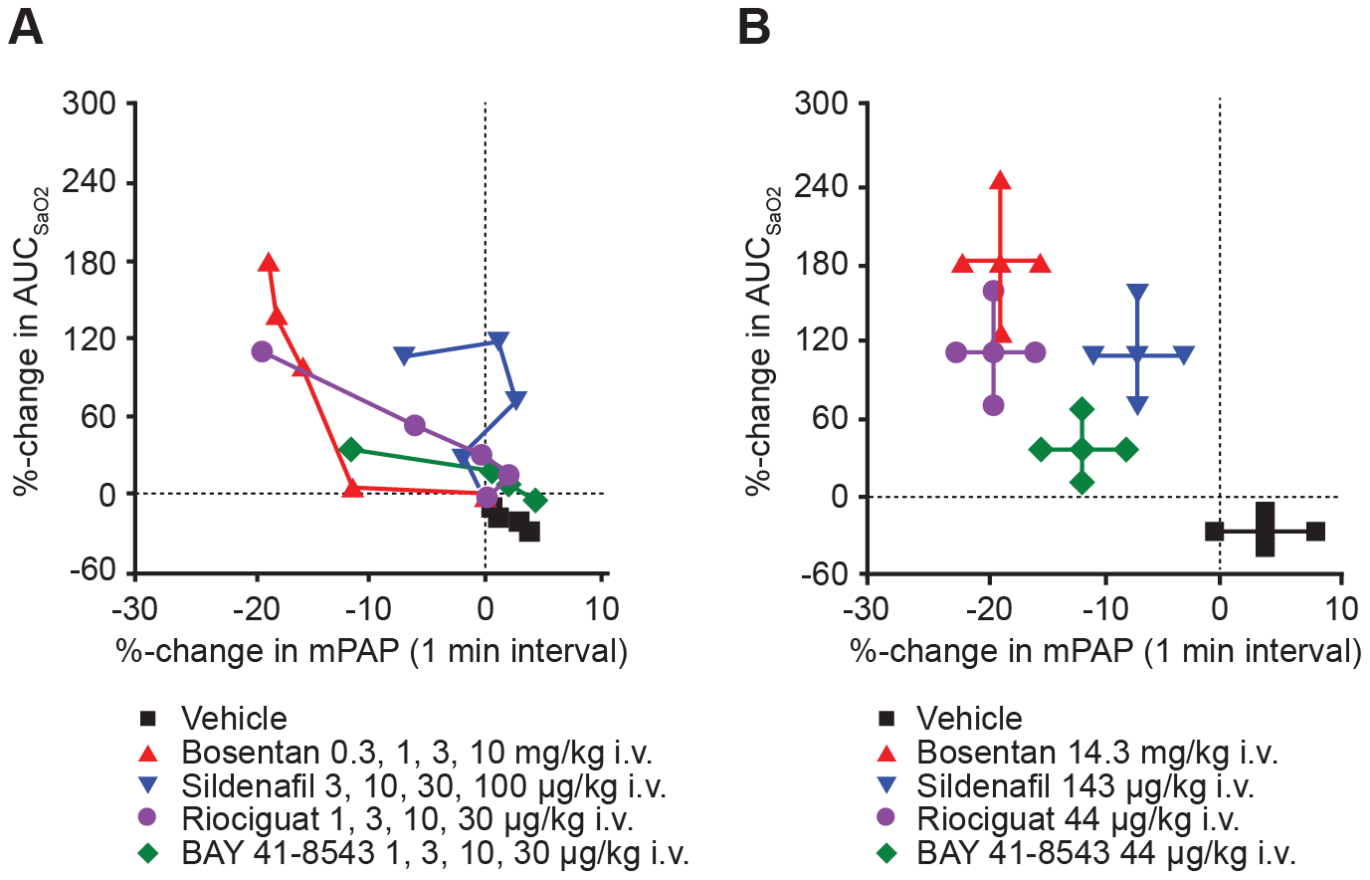
Vasodilator capacity to decrease maximal hypoxic mPAP and AUC_SaO2_. Vasodilator capacity to decrease maximal hypoxic mPAP (positive treatment effect) and AUC_SaO2_ (unwanted desaturation effect) based on (A) pooled effects at all dose levels tested (N = 6, four doses per group) and (B) at each drug's maximal effective dose (i.e., cumulative dose) (mean ± SEM, N = 6, doses as indicated). *P<0.05 vs vehicle. AUC_SaO2_, area under the SaO_2_ curve; SaO_2_, arterial oxygen saturation of hemoglobin; mPAP, mean pulmonary artery pressure; SEM, standard error of the mean.

To compare the risk:benefit ratio of the different vasodilator mechanisms, we evaluated changes in maximal hypoxic PAP on the one hand and unwanted desaturation on the other. Thus, we compared the mean% changes in maximal hypoxic mPAP and associated% changes in AUC_SaO2_ independent of the administered doses (Fig. 8). Vice versa, we compared mean% changes in maximal AUC_SaO2_ increments with associated% changes in mPAP. Across the groups, the overall estimated correlation between both co-primary endpoints (% change in mPAP and% change in AUC_SaO2_) was 0.35, commonly considered a medium rather than a small correlation.

With the final dose step (Cycle 7), disparities between the groups were marked (Fig. 8B), and all treated groups were statistically different from the vehicle group at the 5% level.

For the preceding Cycles 5 and 6 (i.e., second and third dose steps, respectively), differences in the co-primary endpoints between the groups were also statistically significant at the 5% level. This also holds true for each parameter separately (with the exception of% change in increased AUC_SaO2_ at Cycle 5 with a p-value of 7.1%).

Plasma concentrations of each drug 30 min after exposure to cumulative intravenous doses are shown in Supplementary Figure S1. As expected, the wide range of doses chosen resulted in exponential systemic exposure to each vasodilator, which appeared linear on the logarithmic scale.

## Discussion

PH entails a group of distinct and serious medical conditions in which an increase in pulmonary vascular resistance leads to right ventricular overload and, eventually, to right ventricular failure and death [13]. The primary forms of PH (group 1 PH, i.e. without underlying lung disease [14]) carry a poor prognosis without treatment [15]–[19]. Even with the use of a number of approved drugs (prostanoids, endothelin antagonists, and PDE5 inhibitors) for PAH (group 1 PH), mortality rates in these patients remain high at 14% at 1 year and 39% at 5 years [20], [21]. Furthermore, under standard of care approximately 50% of patients with PAH remain in WHO functional class III [20], [21]. In addition, endothelin antagonists and PDE5 inhibitors are not efficacious in secondary forms of PH, partly because of their propensity to aggravate VQM [6], [7]. For instance, through their unique vasodilating mechanism, these drug classes impaired arterial oxygenation when administered to patients with PH secondary to chronic obstructive airways disease [6], [7], [22]. The mechanisms linking vasodilation to desaturation in patients with secondary forms of PH is complex and depends on several variables (e.g., presence of lung disease, resting state vs. exercise, and specific type of vasodilator) [8].

Accurately measuring VQM is technically demanding (e.g., by the multiple inert gas elimination technique [MIGET]) [23], [24]. Therefore, the goal of this study was to evaluate a simplified approach by assessing hemodynamic effects of pharmacologically distinct vasodilator drug classes under conditions of heterogeneous lung ventilation. The drugs chosen were those previously reported to cause VQM [6], [7], [22] and two sGC stimulators (BAY 41–8543 and riociguat). In this model, the respiratory rate and tidal volume were chosen to optimize the physiological range of arterial blood gases. Inadvertent induction of ventilator-induced lung injury cannot be excluded, although no signs indicating lung injury were observed.

Since desaturation is the most clinically relevant effect of VQM in our animal model, we focused on different parameters that could influence arterial oxygen saturation. This pragmatic approach is also feasible for larger clinical trials since in essence the measurement of SaO_2_ is used as a safety biomarker [11]. The caveat is that a drop in SaO_2_ might be caused by different mechanisms unrelated to VQM (e.g., by a drop in central venous saturation [SvO_2_], e.g., associated with increased oxygen extraction due to reduced CO and/or impaired organ perfusion, changes in hemoglobin content). Therefore, the aforementioned factors SvO_2_ and hemoglobin content were controlled for and were indeed not different between treatment groups. Complex animal models of heterogeneous lung injury (e.g., lavage model, oleic acid, lipopolysaccharide induction) often result in high variability of injury causing high variability of treatment effects. Therefore, to evaluate the influence of different treatment options on arterial oxygenation status, we used a simplified approach. Under anesthesia and artificial ventilation, in each animal we performed seven repetitive cycles of unilateral lung ventilation, resulting in transient increases in mPAP accompanied by a decrease in arterial hemoglobin saturation (SaO_2_). During each unilateral ventilation cycle, mechanisms involved in HPV were activated in our experiment, influenced by increasing concentrations of different vasoactive drugs. Under vehicle conditions, the observed tendency to smaller drops in SaO_2_ during the time course of the experiment, as well as the small increase in mPAP, might be related to progressive lung atelectasis in artificially ventilated anesthetized animals.

Due to these observed time-dependent changes in the model, we compared the effects of different vasodilators to vehicle-treated animals at equivalent time points. Stimulation of sGC may also have significant advantages over PDE5 inhibition. Owing to their NO-independent mode of action, sGC stimulators could be effective even when NO production is severely compromised or absent; by contrast, PDE5 inhibitors merely prevent the degradation of cGMP. When NO signaling is disrupted in PH, the production of cGMP may be severely limited, and the preservation of low cGMP levels by PDE5 inhibitors may be ineffective. Moreover, when PDE5 is inhibited, the activity of other PDEs may compensate for it [10]. With NO being an endogenous trigger for the HPV mechanism, it might be possible that sGC stimulators enhance the physiological role of NO in a more physiological way than PDE5 inhibitors, which only prevent cGMP degradation and might be ineffective with low cGMP levels. Therefore, PDE5 inhibitors and sGC stimulators might have different outcomes in VQM assessments.

As expected, all vasodilators induced a dose-dependent decrease in mPAP versus control under normal ventilation but also decreased hypoxia induced mPAP increases during unilateral ventilation cycles. Here, the relation of decreases observed on BP, mPAP, and maximal hypoxic PAP varied by vasodilator type. Regarding mPAP and maximal hypoxic PAP, bosentan and riociguat were more effective than sildenafil. As depicted in Fig. 5, all systemically applied vasodilators dose-dependently enhanced desaturation during unilateral ventilation cycles, as shown by the increase in the desaturation area. In comparison with bosentan and sildenafil, both sGC stimulators had a less pronounced effect on arterial saturation under our experimental conditions. In contrast to observed differences in desaturation potential, all vasodilators (independent of their mechanism) increased CO to a similar extent. In separate animals, to avoid interference with continuous arterial SaO_2_ measurements, arterial and venous blood samples were drawn. We did not observe significant differences in arterial or venous partial O_2_ pressure by treatment group at baseline or during the course of the experiment. A preload dependence of BP caused by a different effect of the vasodilators on left ventricular end diastolic pressure (LVEDP) can be ruled out. In all groups a similar reduction in LVEDP was observed (data not shown). Right ventricular end diastolic pressure was not measured during the experiments. Since we controlled for most physiologic parameters that affect arterial oxygenation (e.g., FiO_2_, SvO_2_, hemoglobin content), we hypothesize that observed differences in desaturation potential between sGC stimulators, the PDE5 inhibitor sildenafil, and the endothelin antagonist bosentan could be related to differences in VQM potential of these drugs.

When we evaluated all compounds regarding their intended effects (reduction of hypoxic mPAP) and the associated unwanted effect (increase in AUC_SaO2_), the pharmacologic mechanisms of each vasodilator were clearly differentiated. Beside the comparable effects on BP, the drugs reduced hypoxic mPAP to a different extent, with the sGC stimulators riociguat and BAY 41–8543 being equipotent to bosentan. Sildenafil, probably due to its limited mechanism of action by PDE5 inhibition, was less effective when compared with bosentan but also when compared with sGC stimulators. Importantly, the sGC stimulators BAY 41–8543 and riociguat were similarly or even less likely to cause an unwanted decrease in SaO_2_, when compared with bosentan and sildenafil. Nevertheless, to avoid desaturation effects which hint towards a VQM under our experimental conditions, the lowest effective dose should be used in clinical trials for all hemodynamic active compounds and oxygenation might serve as a safety biomarker in patients at risk.

With respect to its clinical relevance, our study has a number of limitations: (i) Unilateral lung ventilation, although an inducer of hypoxemia, is not an exact model of the heterogeneous ventilation that is observed in parenchymal lung disease (e.g. acute respiratory distress syndrome, pulmonary fibrosis, or chronic obstructive pulmonary disease) as the pathology affects the lobe evenly and is not related to inflammatory or fibrotic changes. (ii) This study was conducted under anesthesia and mechanical ventilation so we could not evaluate any influence of spontaneous breathing. (iii) In our acute preclinical model, we were not able to address chronic treatment effects, which may be observed in clinical studies. The different pharmacologic mechanisms were compared based on doses that have similar effects on systemic blood pressure. (iv) In addition, the chronic hemodynamic effects of the drugs in the clinical setting differ from the hemodynamic effects in this acute study under anesthetized conditions. Nevertheless, the intravenous pharmacokinetics of all of these drugs are very well characterized. The approximately linear increases in concentration shown here indicate that the plasma concentration increases with each additional dose; this may also occur at the drug target site. The peak concentrations at the start of each additional dose are also likely to be considerably higher than those measured, coming close to the target ranges for human concentrations (bosentan: 500–1000 µg/L [25], sildenafil: 50–150 µg/L [26], riociguat: 70–200 µg/L [27], BAY 418543: experimental compound, human concentration not known). In this study we therefore show that the desaturation potential of all these drugs increases with increasing dose, and that the drugs show differences in their desaturation effects at comparable pharmacologically effective doses (with respect to BP lowering).

With respect to their VQM potential, the various vasodilators could be differentiated. A goal of further research might be to evaluate new vasodilatory mechanisms or treatment options to minimize VQM and maximize efficacy. Agents with lower risk for VQM could be therapeutically advantageous in patients with secondary forms of PH. Future clinical investigations will confirm these findings in the clinical setting.

In conclusion, although each drug class had comparable effects on BP, the reduction in hypoxic mPAP was greatest with the sGC stimulators and bosentan. Furthermore, compared with bosentan and sildenafil, both sGC stimulators were similarly or even less likely to cause an unwanted decrease in SaO_2_.

## Supporting Information

Figure S1 **Plasma levels of compounds after intravenous bolus applications.** Plasma levels are determined 30 min after cumulative applications (mean ± SEM, N = 6). SEM, standard error of the mean.
(TIF)
