# Supplementary figures and images for: Effects of Different Pulmonary Vasodilators on Arterial Saturation in a Model of Pulmonary Hypertension
Source: PLoS One. 2013 Aug 28;8(8):e73502. doi: 10.1371/journal.pone.0073502 (PMC3756006; doi:10.1371/journal.pone.0073502)

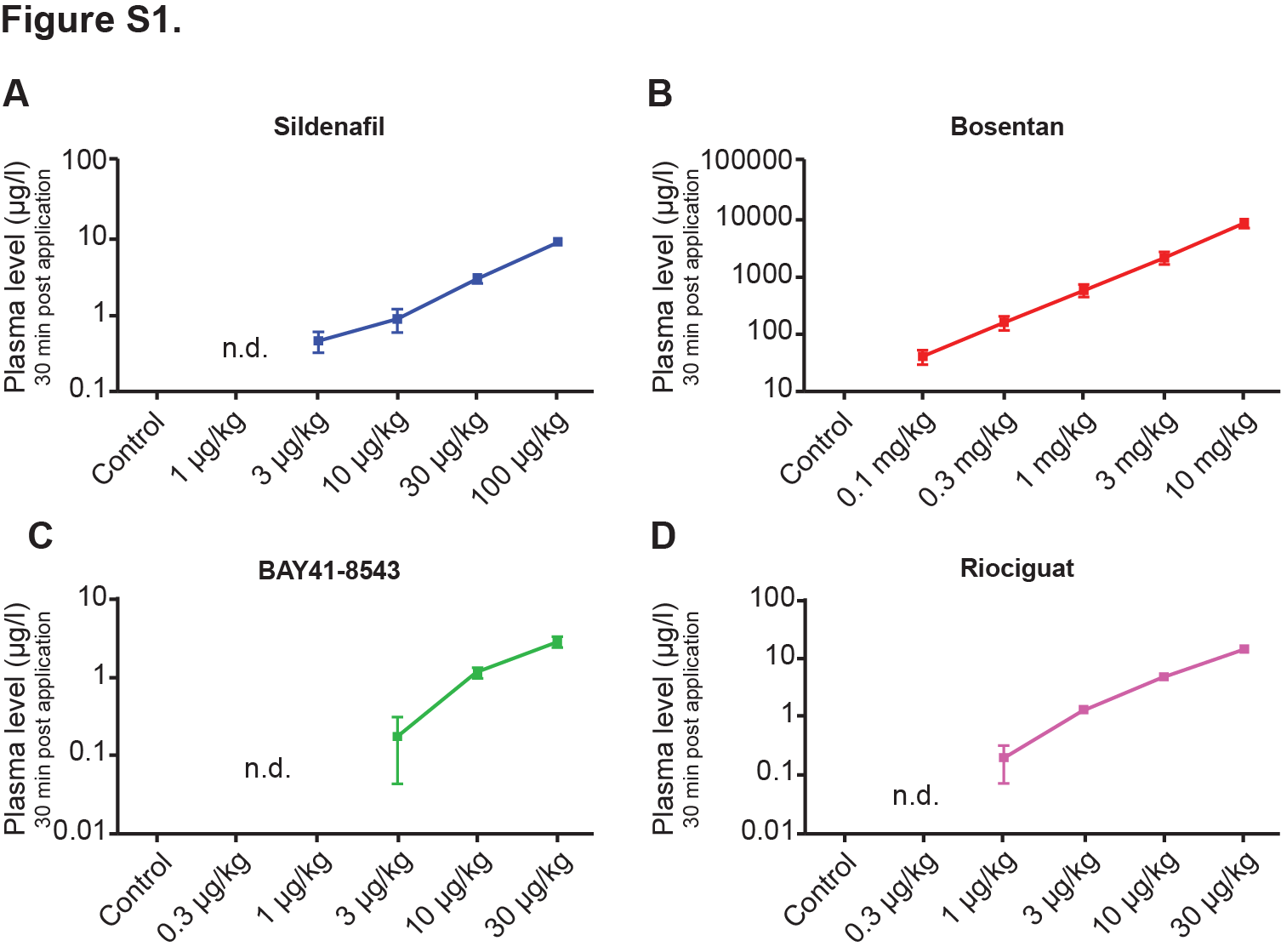

Supplement: Figure S1 — Plasma levels of compounds after intravenous bolus applications. Plasma levels are determined 30 min after cumulative applications (mean ± SEM, N = 6). SEM, standard error of the mean. (TIF) [file pone.0073502.s001.tif]
